# Successful Endoscopic Resection of Primary Rectal Mucosa-Associated Lymphoid Tissue Lymphoma by Endoscopic Submucosal Dissection: A Case Report

**DOI:** 10.3389/fmed.2021.715256

**Published:** 2021-09-10

**Authors:** Jian Han, Zhe Zhu, Chao Zhang, Hua-ping Xie

**Affiliations:** ^1^Department of Gastroenterology, Tongji Hospital of Tongji Medical College, Huazhong University of Science and Technology, Wuhan, China; ^2^Herbert Irving Comprehensive Cancer Center, Columbia University, New York, NY, United States; ^3^Department of Pathology, Tongji Hospital of Tongji Medical College, Huazhong University of Science and Technology, Wuhan, China

**Keywords:** colonoscopy, endoscopic submucosal dissection, endoscopic mucosal resection, mucosa-associated lymphoid tissue lymphoma, rectum

## Abstract

Mucosa-associated lymphoid tissue (MALT) lymphoma arises in extra-nodal sites from the malignant transformation of B lymphocytes that are mainly triggered by infection or autoimmune process. MALT lymphoma is frequently detected in the gastrointestinal tract. As the causal relationship between Helicobacter pylori (H. pylori) infection and gastric MALT lymphoma, it was well-established that early-stage gastric MALT lymphoma could be cured by H. pylori eradication, and about 50–95% of cases achieved complete response with anti-H. pylori treatment. Compared to the stomach which is the most involved site due to the high prevalence of H. pylori infection, the colorectum is rarely affected. Primary rectal MALT lymphoma is a rare malignancy, and there are no specific therapeutic strategies so far. Here we report a case of rectal MALT lymphoma successfully resected by endoscopic submucosal dissection (ESD). ESD serves as a novel strategy to cure small localized rectal MALT lymphomas to avoid unnecessary surgery or chemo-radiotherapy.

## Introduction

MALT lymphoma, classified as an indolent non-Hodgkin's B-cell lymphoma, arises in extra-nodal sites from the malignant transformation of B cells that are mainly triggered by infection or autoimmune process (1–3). Although it might exist in different organs such as the salivary gland, thyroid gland, breast, lung, bladder, skin and orbit, MALT lymphoma is most frequently detected in the gastrointestinal tract (2). Compared to the stomach which is the most involved site due to the high prevalence of H. pylori infection, the colorectum is rarely affected. The pathogenesis of colorectal MALT lymphoma may be associated with microorganisms colonized in the colorectum as reported in several studies (1, 4, 5). Surgical resection, radiotherapy or chemotherapy serve as therapeutic options in the treatment of colorectal MALT lymphomas (6). With the development of technology, ESD emerges as a new therapeutic strategy for colorectal MALT lymphomas as it is regarded as a novel method to cure early gastrointestinal carcinomas and submucosal tumors nowadays. Here we report a case of small rectal MALT lymphoma which is curatively resected by ESD.

## Case Description

**Figure 1:**
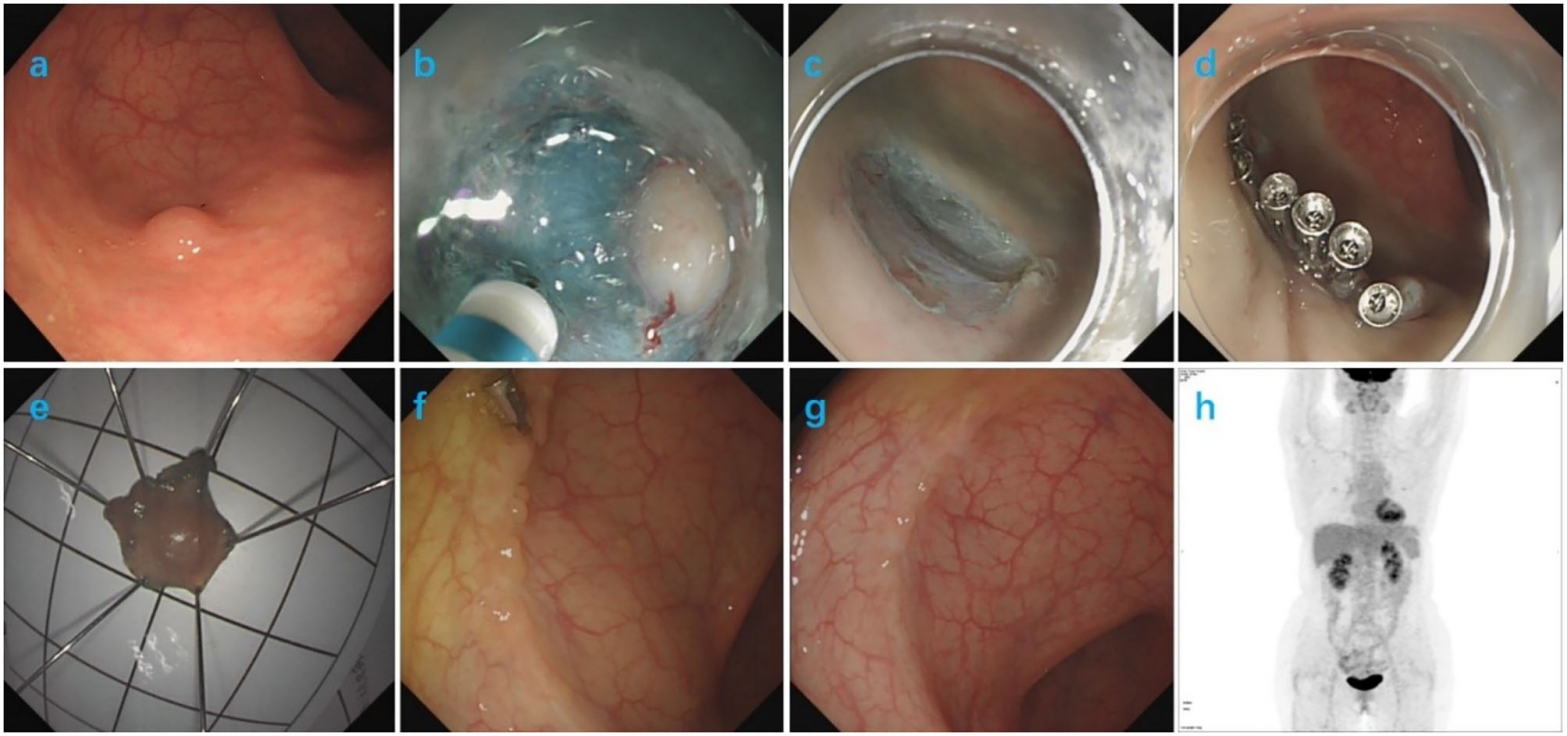
**(a)** Colonoscopy showed a slightly yellowish, submucosal tumor-like 5-mm protrusion in the rectum. **(b–e)** The procedure of ESD. **(f)** Three months after ESD, a follow-up colonoscopy showed complete resolution of the elevated lesion and a residual titanium clip. **(g)** Nine months after ESD, a follow-up colonoscopy showed complete resolution of the elevated lesion. **(h)** PET/CT revealed no evidence of malignancy in the whole body after ESD.

**Figure 2:**
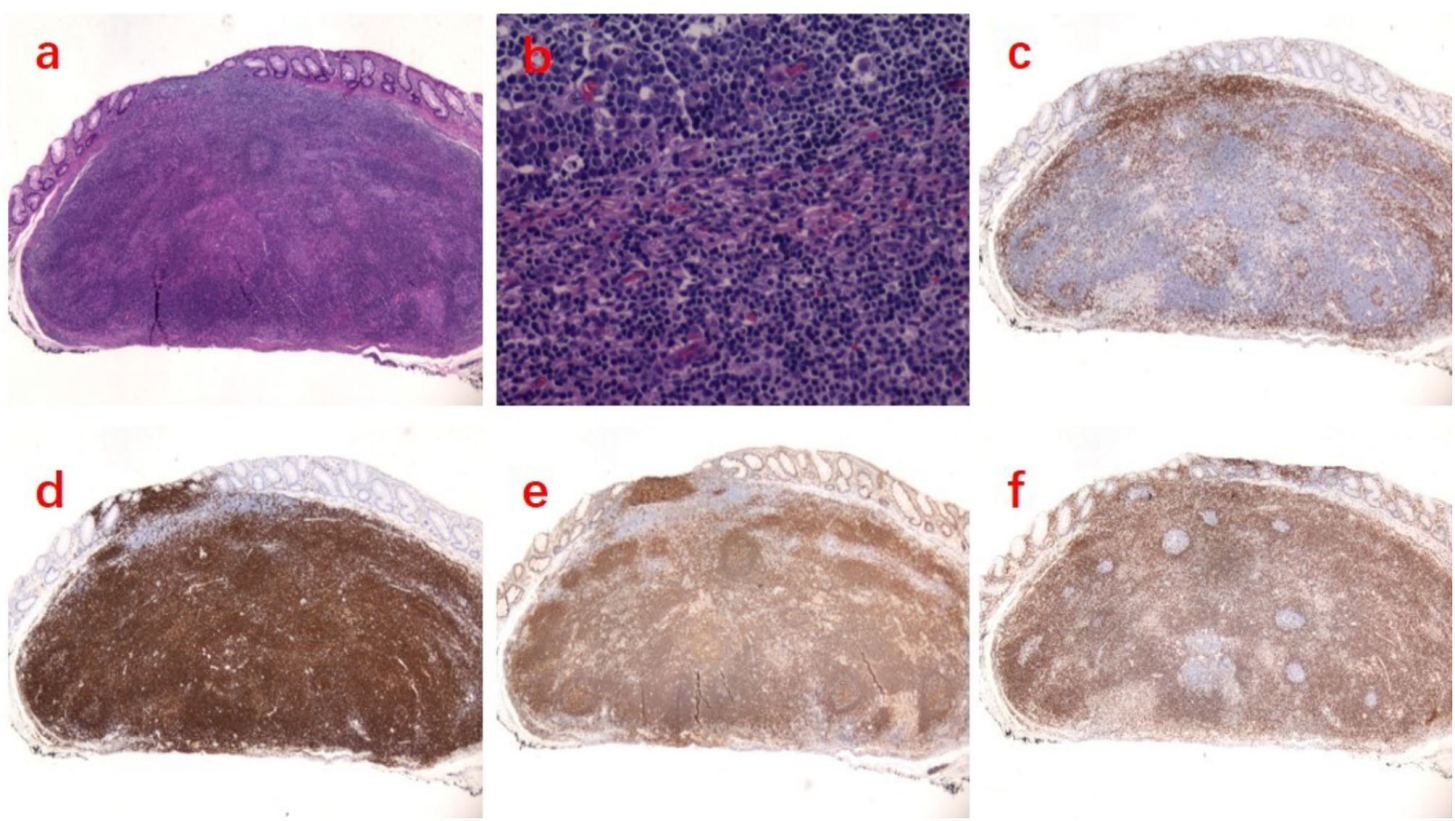
Histopathologic examination revealed a MALT lymphoma of the rectum. **(a)** Hematoxylin-eosin (HE) staining ×20. **(b)** HE staining ×200. **(c)** Immunohistochemistry (IHC) was negative for CD3. **(d)** IHC was positive for CD20. **(e)** IHC was positive for CD21. **(f)** IHC was positive for Bcl-2.

An asymptomatic 58-year-old female patient was admitted to our hospital for routine colonoscopy in 2018. She had no previous history of malignancy or other diseases. A slightly yellowish 5-mm protrusion was detected in the rectum, resembling a submucosal tumor (Figure 1a). The ^13^C urea breath test was negative for H. pylori. Blood routine, urine routine, routine fecal and occult blood, blood biochemistry tests, immune indexes and infection indexes were all within normal ranges. The white light image of the lesion indicated a possibility of a neuroendocrine tumor and therefore we resected it using ESD (Figures 1b–e). After marking the resection borders of the lesion, a submucosal cushion was created by injecting a mixture of saline solution, methylene blue, and adrenaline. A total circumferential incision and submucosal excision and dissection was performed by using a DualKnife (Olympus). Additionally, we performed endoclip closure for mucosal defect after ESD. No complication occurred during or after ESD. The histopathological findings of the ESD sample from the rectal lesion confirmed the diagnosis of a rectal MALT lymphoma, with diffuse infiltration of small-sized lymphoid cells, which were positive for CD20, Bcl-2, CD21, CD35 (partial), kappa (partial) and lambda (partial), but negative for CD3, CD5, CD10, and cyclin D1. The resected margin was clean both horizontally and vertically (Figure 2). PET/CT demonstrated negative evidence of malignancy in the whole body after ESD (Figure 1h). The endoscopic follow-up at the 3rd month and the 9th month, respectively, after ESD showed no residual or recurrent lesions (Figures 1f,g). The timeline with relevant data from the episode of care was showed in Table 1.

**Table 1:** The timeline with relevant data from the episode of care.

**Timeline**	**Admission day 1**	**Admission day 2**	**Admission day 3**	**5 days after ESD**	**6 days after ESD**	**3 months after ESD**	**9 months after ESD**
Clinical data	The ^13^C urea breath test was negative. Blood routine, urine routine, fecal routine, biochemistry tests, immune indexes and infection indexes were all within normal ranges.	A slightly yellowish 5-mm protrusion was detected in the rectum by colonoscopy. The white light image indicated a possibility of a neuroendocrine tumor and we resected it by ESD. No complication occurred during ESD.	The patient was discharged from the hospital without complication after ESD.	The pathological findings of the rectal lesion confirmed the diagnosis of a MALT lymphoma.	PET/CT demonstrated negative evidence of malignancy in the whole body after ESD.	The endoscopic follow-up at the 3rd month after ESD showed no residual or recurrent lesions.	The endoscopic follow-up at the 9th month after ESD showed no residual or recurrent lesions.

## Discussion

Rectal MALT lymphoma is a rare malignant disease with limited reports in the literature and there is lack of definite treatment strategies (6). Due to the close association between gastric MALT lymphoma and H. pylori infection, eradication of H. pylori is strongly recommended for the treatment of gastric MALT lymphoma, even for patients with negative test of H. pylori (3). Actually, a few cases of colorectal MALT lymphomas were previously reported to benefit from the eradication of H. pylori (7, 8). However, 16 of 17 patients with extra-gastric MALT lymphomas were recently reported without regression of lymphoma with a follow-up of 14 months after H. pylori eradication, which indicated that H. pylori eradication was ineffective for treatment of extra-gastric MALT lymphomas (4). Although surgical resection, radiotherapy or chemotherapy can cure or regress colorectal MALT lymphomas, endoscopic mucosal resection (EMR) was also reported to cure small colorectal MALT lymphomas (6). Compared to EMR, ESD is superior because it allows en bloc resection and accurate histological examination (9). Choi reported that ESD successfully treated residual rectal MALT lymphomas after EMR, and Akasaka reported a case of complete endoscopic resection of a rectal MALT lymphoma by ESD (6, 10). To the best of our knowledge, this is the third case report of resection of rectal MALT lymphoma by ESD. Although rectal MALT lymphoma is a rare disease, the appropriate evaluation and proper treatment option might benefit the patients. ESD provides a novel therapeutic strategy for small localized primary rectal MALT lymphomas to avoid unnecessary surgical resection or chemo-radiotherapy. Endoscopic resection can be recommended for properly selected patients with localized and endoscopically resectable small primary rectal MALT lymphomas as it is effective and minimally invasive, and close follow-up after ESD is needed. This case report adds to the body of literature to the effectiveness of ESD in the management of a number of early gastrointestinal cancers. Since rectal MALT lymphomas are rare, their optimal management remains unclear. Now we have more evidence to support the use of ESD in the management of such tumors. More data about this disease is urgently required to provide better insight and treatment strategies.

## Data Availability Statement

The raw data supporting the conclusions of this article will be made available by the authors, without undue reservation.

## Ethics Statement

Ethical review and approval was not required for the study on human participants in accordance with the local legislation and institutional requirements. The patients/participants provided their written informed consent to participate in this study. Written informed consent was obtained from the individual(s) for the publication of any potentially identifiable images or data included in this article.

## Author Contributions

JH and H-pX designed the study. CZ performed the pathologic analysis. H-pX performed ESD and was responsible for the revision of the manuscript. JH and ZZ wrote the original draft. All authors read and approved the manuscript.

## Conflict of Interest

The authors declare that the research was conducted in the absence of any commercial or financial relationships that could be construed as a potential conflict of interest.

## Publisher's Note

All claims expressed in this article are solely those of the authors and do not necessarily represent those of their affiliated organizations, or those of the publisher, the editors and the reviewers. Any product that may be evaluated in this article, or claim that may be made by its manufacturer, is not guaranteed or endorsed by the publisher.
